# Evaluation of a multimodal diagnostic algorithm for prediction of cognitive impairment in elderly patients with dizziness

**DOI:** 10.1007/s00415-024-12403-3

**Published:** 2024-05-03

**Authors:** K. Felfela, N. Jooshani, K. Möhwald, D. Huppert, S. Becker-Bense, F. Schöberl, R. Schniepp, F. Filippopulos, M. Dieterich, M. Wuehr, A. Zwergal

**Affiliations:** 1 German Center for Vertigo and Balance Disorders (DSGZ), LMU University Hospital, LMU Munich, Marchioninistr. 15, 81377 Munich, Germany; 2 Department of Neurology, LMU University Hospital, LMU Munich, Munich, Germany; 3 Cluster for Systems Neurology, SyNergy, Munich, Germany

**Keywords:** Clinical vestibular testing, Gait assessment, Neuroimaging, Dizziness, Cognitive impairment, Dementia

## Abstract

**Background:**

The current diagnostic workup for chronic dizziness in elderly patients often neglects neuropsychological assessment, thus missing a relevant proportion of patients, who perceive dizziness as a subjective chief complaint of a concomitant cognitive impairment. This study aimed to establish risk prediction models for cognitive impairment in chronic dizzy patients based on data sources routinely collected in a dizziness center.

**Methods:**

One hundred patients (age: 74.7 $\pm$ 7.1 years, 41.0% women) with chronic dizziness were prospectively characterized by (1) neuro-otological testing, (2) quantitative gait assessment, (3) graduation of focal brain atrophy and white matter lesion load, and (4) cognitive screening (MoCA). A linear regression model was trained to predict patients’ total MoCA score based on 16 clinical features derived from demographics, vestibular testing, gait analysis, and imaging scales. Additionally, we trained a binary logistic regression model on the same data sources to identify those patients with a cognitive impairment (i.e., MoCA < 25).

**Results:**

The linear regression model explained almost half of the variance of patients’ total MoCA score (*R*^2^ = 0.49; mean absolute error: 1.7). The most important risk-predictors of cognitive impairment were age (*β* = − 0.75), pathological Romberg’s sign (*β* = − 1.05), normal caloric test results (*β* = − 0.8), slower timed-up-and-go test (*β* = − 0.67), frontal (*β* = − 0.6) and temporal (*β* = − 0.54) brain atrophy. The binary classification yielded an area under the curve of 0.84 (95% CI 0.70–0.98) in distinguishing between cognitively normal and impaired patients.

**Conclusions:**

The need for cognitive testing in patients with chronic dizziness can be efficiently approximated by available data sources from routine diagnostic workup in a dizziness center.

**Supplementary Information:**

The online version contains supplementary material available at 10.1007/s00415-024-12403-3.

## Introduction

Current classifications of vestibular disorders almost exclusively list peripheral neuro-otological disorders such as bilateral vestibulopathy as a cause of chronic dizziness [1, 2], while central or higher vestibular network pathologies are implicated less often [3]. Accordingly, routine workup of dizzy patients presently focusses on quantification of function of peripheral vestibular afferents and reflexes (such as the vestibulo-ocular reflex). However, especially in an elderly population, a relevant proportion of dizzy patients has no sufficient explanation of their symptoms based on peripheral vestibular function tests only. Recently, a syndrome called Dizziness in Cognitive Impairment (DCI) was shown to account for up to one third of cases with chronic dizziness in patients above the age of 60 years [4]. In this syndrome, a specific presumably neurodegenerative pattern with a selective effect on parieto-insular multisensory vestibular and cognitive networks was identified as the potential pathophysiological correlate. Several previous studies have reported a prevalence of dizziness of 30–50% in patients with various dementia syndromes [5, 6]. Despite this obvious interrelation, the simultaneous appraisal of dizziness and cognitive impairment in clinical route diagnostics is often neglected [6, 7]. The primary reasons for this shortcoming may be firstly that routine cognitive screening of elderly dizzy patients might be too time-consuming and secondly that there are to date no validated questionnaires or rating scales available, which are sufficiently sensitive to detect both vestibular and cognitive complaints [7].

A potential solution for this drawback may be to combine data sources from clinical routine testing to establish prediction models for cognitive impairment in chronic dizziness. The most promising candidates are likely demographic factors (mostly age) [8], vestibular function testing [9], gait and posture assessment, as well as neuroimaging [4]. Studies in age cohorts, for example, were able to link measurements of postural stability and gait with cognitive function and even neurodegenerative alterations [10, 11]. Growing evidence indicates that cognitively impaired patients display an altered gait pattern including reduced speed especially in dual-task conditions as well as an increased variability of time-related gait parameters [11]. These gait pattern changes can even precede cognitive decline and are therefore considered as early risk markers for dementia [12].

The main aim of the current study was thus to assess the predictive value of routine data sources, such as demographic parameters, vestibular tests, gait and posture assessment, as well as neuroimaging separately and in combination, for the prediction of cognitive function and impairment in patients with chronic dizziness. Using this approach, we aimed to identify readily available risk markers for cognitive dysfunction in patients with chronic dizziness, which should indicate the need for a dedicated follow-up neuropsychological testing. This could help to improve current standards of practice in the differential diagnosis of elderly patients with chronic dizziness.

## Materials and methods

### Participants and study design

**Fig. 1:**
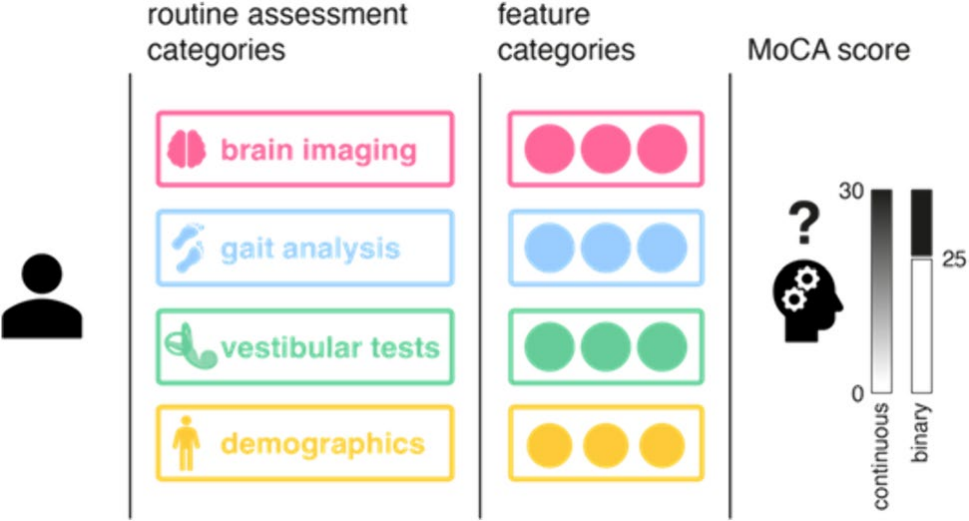
Illustration of the study concept. The main objective of the current study was to evaluate single and combined routine assessment categories in chronic dizzy patients for their potential to predict cognitive function and classify patients as cognitively unimpaired and impaired individuals. For this purpose, linear and binary logistic regression analyses were performed based on feature categories to predict the absolute MoCA score of individual patients (continuous prediction) and to identify those patients with an explicit cognitive impairment (MoCA < 25; binary classification)

In this prospective cohort study, 100 patients (age > 60 years, age: 74.7 ± 7.1 years, 41.0% women), who presented to the German Center for Vertigo and Balance Disorders (LMU Munich, Germany) with the chief complaint of chronic dizziness, vertigo or balance disorders (thereafter referred to under the umbrella term “dizziness”, detailed symptoms in supplement), were recruited consecutively over a period of 3 months and were thoroughly characterized by (1) neuro-otological examination (including video head impulse testing (vHIT), caloric testing, Romberg’s test); (2) in-laboratory quantitative gait assessment (GAITRite^®^ sensor carpet, functional gait assessment—FGA, timed-up-and-go test—TUG); (3) grading of routine brain magnetic resonance (MR) or computer tomography (CT) images regarding white matter lesion load and focal brain atrophy using established visual rating scales; and (4) Montreal Cognitive Assessment (MoCA) score as a cognitive screening test (Fig. 1). Neuro-otological diagnoses were made following the established International Classification of Vestibular Disorders (ICVD) for unilateral or bilateral vestibulopathy, Menière's disease, benign paroxysmal positional vertigo, vestibular paroxysmia, and vestibular migraine [1].

### Neuro-otological testing

Experienced technicians conducted horizontal vHIT testing using a lightweight video-oculography device (EyeSeeCam^®^, Fürstenfeldbruck, Germany) with an integrated digital high-speed camera. The vHIT gain was calculated as a ratio of eye per head acceleration at 60 ms to evaluate the function of the vestibulo-ocular reflex (VOR) in the high-frequency range. Additionally, for caloric testing both ears’ external auditory canals were irrigated once with 20 ml cold (30 °C) and once with 20 ml warm (44 °C) water. The maximum slow phase velocity (mSPV in °/s) of caloric nystagmus was determined to quantify VOR function in the low-frequency range. A stabilometer platform was used to conduct the posturographic examination (Kistler^®^, Winterthur, Switzerland) in accordance with previously established protocols [13]. The Romberg’s quotient (RQ) (i.e., the ratio of postural sway path with eyes closed to that with eyes open) was then calculated in anterior–posterior (AP) and medio-lateral (ML) axes, and was considered pathological if the ratio in any of the two axes was $\ge$ 2 [14]. Alternatively, in patients without a posturography analysis (*n* = 42), an increase of body sway in Romberg’s test with eyes closed was clinically rated as pathological by experienced neuro-otologists. Ocular and cervical vestibular evoked myogenic potentials (oVEMPs and cVEMPs) were not performed routinely.

### Quantitative gait analysis

Gait patterns were assessed using a 6.7 m long pressure-sensitive gait carpet (GAITRite^®^, CIR Systems, Havertown, USA) with a sampling frequency of 120 Hz during walking at preferred speed and during walking with a cognitive dual task (serial 7). Patients walked in total four times across the carpet in each gait condition to collect enough strides for further analysis. Additionally, cognitive dual task costs during walking were evaluated as follows: first, by the percentage change of speed during dual task walking compared to single task walking (locomotor costs), and second, by the change of frequency for correct numerical operations during dual task walking vs. sitting (cognitive costs). For the timed-up-and-go (TUG) test, patients were seated on a chair and asked to stand up on verbal command, walk 3 m, turn around, walk back to the chair and sit down. All subjects also received a functional gait assessment (FGA) consisting of a 10-item clinical gait performance evaluation with an overall score ranging from 0 to 30, with 30 being the best possible score.

### Neuroimaging: visual rating scales

In the routine brain MR- or CT-images, white matter lesion load and focal brain atrophy were assessed using the following established visual rating scales: the FAZEKAS scale for cerebral microvascular lesions (0–3) [15], the global cortical atrophy—frontal scale (GCA-F) (0–3), mesiotemporal atrophy (MTA)-scale (0–4), KOEDAM-scale for parieto-occipital atrophy (0–3) [16, 17], the insula opercular atrophy (IOA) scale (0–3) [4]. Focal brain atrophy was dichotomized for further data analysis into normal and abnormal based on suggestions from previous studies, i.e. scores of ≥ 2 in GCA-F, KOEDAM, IOA or MTA-scale (for patients < 75 years) and scores of ≥ 3 in MTA-scale (for patients ≥ 75 years) [18–20]. In the same way, FAZEKAS grades ≥ 2 were interpreted as abnormal [21].

### Neuropsychological tests and symptom questionnaires

A standardized neuropsychological screening test using MoCA was performed with all participants. Patients with total MoCA scores < 25 were classified as cognitively impaired [22]. The Beck Depression Inventory II (BDI-II) [23], the European Quality of Life-5 Dimensions-5 Levels (EQ-5D-5L) questionnaire [24], and the Dizziness Handicap Inventory (DHI) [25] were assessed to evaluate the potential effect of confounders such as depression, perceived health-related disability and symptom severity on cognitive performance.

### Statistical analysis

Descriptive statistics and univariate analysis using a combination of parametric (i.e., ANCOVA and Student’s *t* test) and non-parametric tests (i.e., Quade- and Chi^2^-tests) were performed in SPSS (Version 27.0, IBM Corp., USA) to test for differences in clinical features between cognitively impaired (MoCA < 25) and normal patients. Age was considered as a covariate to control for age differences between both groups. Normal distribution was assessed using Q–Q plot diagrams and histograms. Results were considered significant at *p* < 0.05. In general, only patients with a complete data set were included in the final analysis. For single missing data (about 1% of all data) multiple imputation was applied.

Linear and logistic regression was performed to generate models for the prediction of cognitive function and impairment (i.e., MoCA score) or a classification of cognitively impaired vs. normal patients, respectively. Regression analysis was done using python programming language Version 3.8 and the open-source module scikit-learn for statistical testing and machine learning. Linear regression with regularization was performed using Least Absolute Shrinkage and Selection Operator (LASSO), which penalizes the regression model via L1-norm and in effect shrinks the effect of non-contributing features to zero. A tenfold nested cross-validation (CV) was performed for model selection and hyperparameter tuning. The complete dataset was initially split into training (80%) and test (20%) sets. Starting from the training set, the outer CV measured the performance of each model, while the inner was used to tune the model hyperparameters by minimizing the mean squared error (MSE) in each fold of the outer CV. The model with the best performance (i.e., minimum MSE) was then trained on the whole training set in a tenfold CV and its performance was assessed on the remaining test set.

Logistic regression with L2-norm regularization was performed for binary classification of cognitively impaired vs. normal patients. Model performance was tested using a tenfold CV and evaluated by the area-under-the-curve (AUC) of a receiver-operating-characteristic (ROC). Binary classification models were trained and evaluated separately for 4 different feature sets (demographics, clinical vestibular testing, gait parameters, imaging parameters) as well as for the entire feature set (age, gender, cardiovascular risk factors (CVRF), Romberg sign, caloric excitability, vHIT gain, preferred speed, dual task speed costs, cognitive costs, TUG, FGA, FAZEKAS, GCA-F, MTA, KOEDEM, IOA scale).

## Results

### Patient characteristics and clinical features

**Fig. 2:**
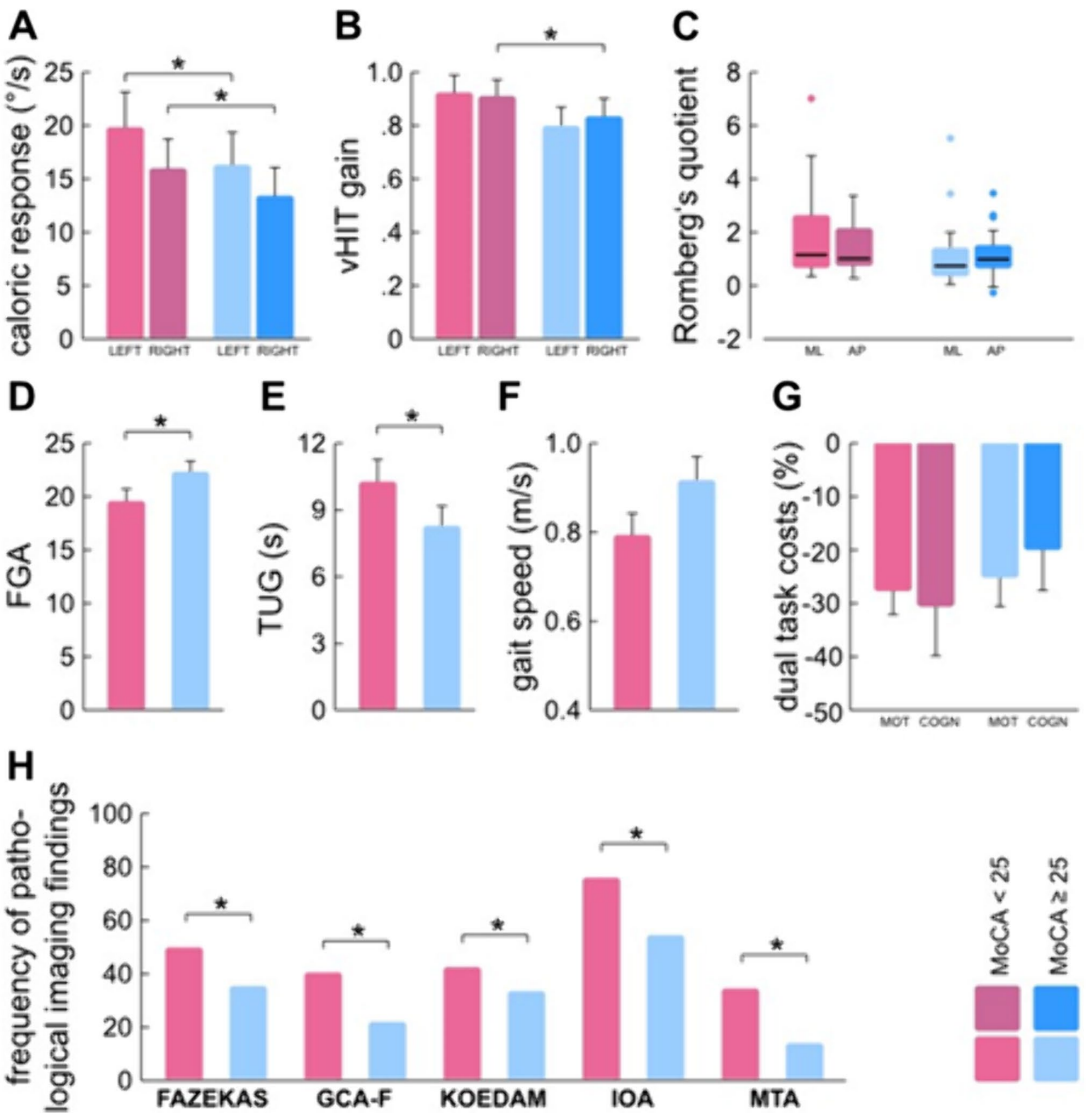
Features from vestibular tests, gait and brain imaging between MoCA groups. Average caloric response (**A**), vHIT gain (**B**), Romberg’s quotient (**C**), functional gait assessment (FGA) (**D**), timed-up-and-go test (TUG) (**E**), preferred gait speed (**F**), dual task locomotor (MOT) and cognitive (COGN) costs (**G**), frequency of pathological imaging findings (**H**). *p < 0.05. *ML* medio-lateral, *AP* anterior–posterior, *GCA_F* global cortical atrophy-frontal scale, *IOA* insula opercular atrophy scale, *MTA* mesiotemporal atrophy scale

While the cognitively impaired group was older than the cognitively healthy group (*p* < 0.001, Student’s *t* test), no significant differences were observed regarding other collected demographic features (Table 1). Neuro-otological diagnoses as defined by ICVD (see methods) were significantly more prevalent in the group of patients with MoCA ≥ 25 (Table 1). Peripheral vestibular results, with the exception of the left-sided vHIT gain, differed significantly across the two cognitive groups (mean mSPV right/left: *p* = 0.03/*p* = 0.04, vHIT gain right/left: *p* = 0.017/*p* = 0.268, ANCOVA, vHIT refixation saccades: *p* = 0.028) and tended to be lower towards higher MoCA scores. Patients with cognitive dysfunction also displayed more pronounced balance deficits compared to the cognitively normal group (*p* = 0.017, Chi-square test) (Table 2). The two groups, however, did not differ regarding either walking speed in preferred and dual task conditions or dual task locomotor and cognitive costs (*p* ≥ 0.06, ANCOVA). Cognitively impaired subjects took longer to perform the TUG test and scored less in the FGA than cognitively normal patients (*p* = 0.03, *p* = 0.023, respectively, ANCOVA). Imaging markers also indicated a higher prevalence of pathological cortical atrophy for all lobes and white matter lesion load towards lower MoCA scores (*p* ≤ 0.008, Chi-square test) (see Fig. 2 and Table 2).

**Table 1:** Comparison of demographics and clinical neuro-otological diagnoses among MoCA groups

	MoCA < 25 (*n* = 45)	MoCA $\ge$ 25 (*n* = 55)	*p* value
Age^a^	77.7 $\pm$ 5.0	72.3 $\pm$ 7.6	< 0.001*
Gender F^b^	35.6%	45.5%	0.599
CVRF^c^	1 $\pm$ 1	1 $\pm$ 2	0.356
Neuro-otological diagnosis^b^	31.1%	54.5%	0.034*
Acute unilateral peripheral vestibulopathy	13.3%	14.5%	0.447
Bilateral vestibulopathy	8.9%	25.5%	0.070
Menière's disease	0%	3.6%	0.069
Vestibular migraine	4.4%	3.6%	0.570
Benign paroxysmal positional vertigo	4.4%	7.3%	0.705
Vestibular schwannoma	0%	5.4%	0.068

*p < 0.05

^a^Mean $\pm$ standard deviation, Student’s *t* test (two-sided *p* value)

^b^Frequencies, Chi-square test

^c^Median $\pm$ interquartile (IQ) range, Quade test. Multiple diagnoses were possible

**Table 2:** Comparison of patients’ vestibular testing, in-laboratory gait assessment, imaging markers, across MoCA groups

	MoCA < 25 (*n* = 45)	MoCA $\ge$ 25 (*n* = 55)	*p* value
Vestibular testing^a,b,c^			
Average caloric excitability (in °/s)^a^	17.8 $\pm$ 10.5	15.0 $\pm$ 10.0	0.031*
Right ear mean caloric response (in °/s)^a^	15.8 $\pm$ 10.0	13.2 $\pm$ 10.4	0.030*
Left ear mean caloric response (in °/s)^a^	19.6 $\pm$ 11.5	16.3 $\pm$ 11.3	0.040*
vHIT gain right^a^	93.0 $\pm$ 25.2	80.3 $\pm$ 30.1	0.017*
vHIT gain left^a^	91.1 $\pm$ 25.5	84.0 $\pm$ 31.6	0.268
vHIT refixation saccades total^b^	5 (11.1%)	18 (32.7%)	0.028*
RQ in ML-axis^b^	1.16 $\pm$ 2.03 (*n* = 23)	0.74 $\pm$ 0.94 (*n* = 34)	0.083
RQ in AP-axis^b^	1.02 $\pm$ 1.25 (*n* = 23)	1.00 $\pm$ 0.72 (*n* = 35)	0.349
Pathological Romberg’s sign^c^	66.7%	38.2%	0.017*
In-laboratory quantitative gait assessment^a^			
PS: velocity (in m/s)	0.78 $\pm$ 0.18	0.91 $\pm$ 0.22	0.060
DTC: velocity (in m/s)	0.57 $\pm$ 0.18	0.68 $\pm$ 0.25	0.248
Costs: locomotor	− 27.2 $\pm$ 16.3	− 24.7 $\pm$ 21.8	0.841
Costs: cognitive	− 30.68 $\pm$ 30.94	− 19.88 $\pm$ 27.91	0.349
TUG	10.14 $\pm$ 3.74	8.29 $\pm$ 3.25	0.030*
FGA	19.44 $\pm$ 4.61	22.20 $\pm$ 4.64	0.023*
Imaging^d^			
FAZEKAS	48.9%	34.5%	0.002*
Mean GCA-F	44.4%	23.6%	< 0.001*
Mean KOEDAM	46.7%	36.4%	0.008*
Mean MTA	37.8%	14.5%	0.006*
Mean IOA	84.4%	60%	< 0.001*

*Marks significant results

^a^Mean $\pm$ standard deviation, ANCOVA

^b^Median $\pm$ interquartile range, Quade-test

^c^Frequencies, Chi^2^ test

^d^Frequencies of pathological white matter lesion load (i.e., FAZEKAS ≥ 2) and pathological brain lobe atrophy, Chi^2^ test

### Prediction models

**Fig. 3:**
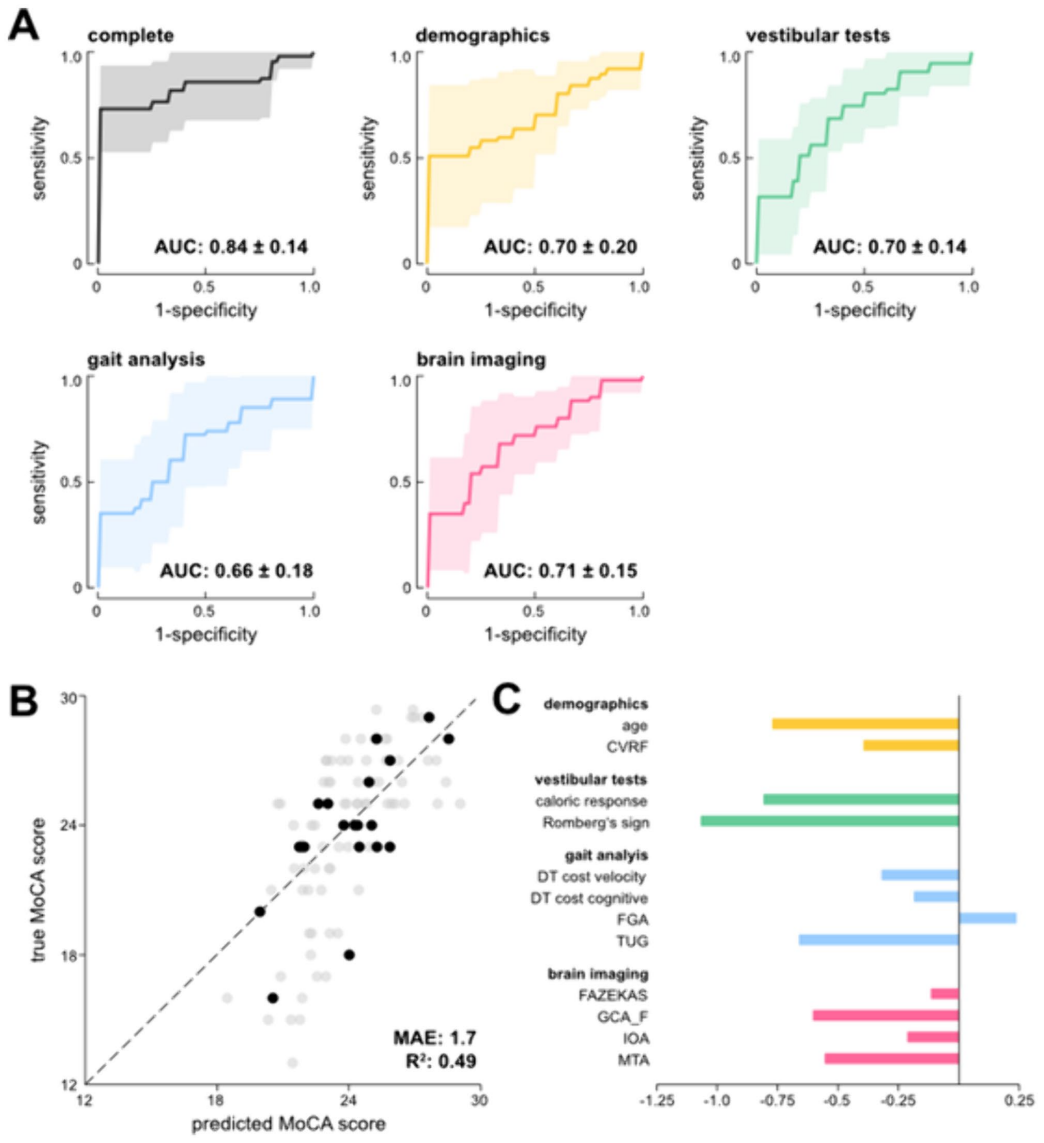
Evaluation of the performance of the regression models for prediction of cognitive impairment. **A** Results from the binary logistic regression analysis to classify cognitively impaired patients (MoCA < 25) with respective ROC-analysis curves (mean ± SD, shaded area) and area under the curve (AUC) outcomes once based on the complete set of clinical features and once considering only features from one category. **B** and **C** Results from the linear regression analysis to predict the total MoCA score of patients. **B** Scatter plot of true vs. predicted MoCA values (grey dots indicate training cases and black dots validation cases). The linear regression model yielded a *R*^2^ value of 0.49 with a mean absolute error (MAE) of 1.7. **C** Standardized coefficient values of the 12 clinical features (stratified by the four clinical categories) included in the linear model. *CVRF* cardiovascular risk factors, *DT* dual task, *FGA* functional gait assessment, *GCA_F* global cortical atrophy-frontal scale, *IOA* insula opercular atrophy scale, *MTA* mesiotemporal atrophy scale, *TUG* timed-up-and-go test

Considering all available clinical features in a binary logistic regression model yielded an AUC of 0.84 (95% CI 0.70–0.98) in distinguishing between MoCA groups < 25 and ≥ 25. Solely considering features from the demographics category yielded an AUC of 0.70 (95% CI 0.50–0.90), from the clinical vestibular testing category an AUC of 0.70 (95% CI 0.56–0.84), from the gait assessment category an AUC of 0.66 (95% CI 0.48–0.84), and from the brain imaging category an AUC of 0.71 (95% CI 0.56–0.86) (Fig. 3A).

All 16 clinical features from the four assessment categories (i.e., demographics, vestibular tests, gait analysis and brain imaging) with at least three features per category were included to establish a linear regression model that should predict the total MoCA score of individual patients. The obtained linear model included 12 clinical features and yielded a *R*^2^ value of 0.49 with a mean absolute error (MAE) of 1.7 and thereby explained nearly 50% of cognitive variance in our cohort (Fig. 3B). Romberg’s sign, caloric response, age and TUG had the highest predictive value, while FAZEKAS, dual task cost cognitive, IOA and FGA had the lowest standardized coefficient values (Fig. 3C).

## Discussion

Dizziness in Cognitive Impairment (DCI) has recently been described as a common syndrome in elderly patients with chronic dizziness, balance problems and instability of stance and gait [4]. However, testing for cognitive impairment is still often neglected in the current diagnostic algorithms for dizzy patients. Therefore, we aimed to establish a prediction model for cognitive function and impairment in elderly patients, based on commonly available clinical features from demographics, vestibular and gait assessment, as well as brain imaging. We demonstrate that prediction models considering features from all four routine assessment categories yielded a high and robust classification of cognitive impairment with an AUC of 0.84 $\pm$ 0.14 and were able to explain about 50% of the cognitive variance in our patient cohort. We further show that considering clinical features from one routine assessment category only, considerably limits model performance. The proposed model could help to appropriately and efficiently select dizzy patients in need of neuropsychological testing and training.

### Rationale behind the model feature selection

The machine learning approach in this study selected features well discussed for their potential in the prediction of the risk of cognitive decline. The parameters with a significant effect in the model ranked from highest to lowest predictive contribution are age > CVRF (demographics), Romberg’s sign > caloric response (vestibular), TUG > dual task cost velocity > FGA > dual task cost cognitive (gait) and GCA-F > MTA > IOA > FAZEKAS (imaging).

Demographic factors such as older age as well as CVRF are associated not only with vascular but also neurodegenerative dementia [26–28]. Previous studies were able, for instance, to show the influence of older age on fluid cognition (i.e., the ability to process and learn new information and adapt to new circumstances) such as processing speed, working memory and executive cognitive function [8].

Vestibular contribution to cognitive functioning has been attracting increasing attention in recent years. For instance, brain networks involved in postural stability such as the cingulo-opercular, fronto-parietal, and somatosensory-motor networks are known to play a substantial role in normal cognitive performance [29]. Therefore, it is plausible that patients with morphological or vascular cortical pathologies within these shared brain networks not only exhibit a dynamic balance disturbance but also cognitive dysfunction, as shown in the recently described DCI syndrome [4]. Kido et al. for example, showed that postural instability is related to pathological cognitive decline and also neurodegenerative diseases [10]. In line with the recent study introducing the DCI syndrome [4], the degree of caloric response in the current study displayed a negative correlation with the MoCA scores (i.e., the lower the caloric response, the higher the MoCA scores). A possible explanation for this observation could be that lower caloric responses filter out patients with clear peripheral vestibular deficits leaving those with central pathologies, which in case of cortical affection of the multisensory vestibular processing network can also manifest with a concomitant cognitive dysfunction [4]. In addition, central pathologies may display a higher caloric response, based on disinhibition of cortico-cerebellar networks. At first sight, the finding of reduced peripheral vestibular function in cognitively unimpaired patients seems to partially contradict the previously reported deficits in visuo-spatial domains in patients with bilateral vestibulopathy [30]. However, these deficits in single cognitive subdomains are rather mild and may not be reflected optimally by MoCA screening. Importantly, the current study does not claim any causality between vestibular test results and the cognitive status, but only reports markers associated with presence of a cognitive impairment.

Gait patterns have also been shown to give insights into cognitive dysfunction and higher cortical and subcortical network pathologies [11]. A complex network of brain regions is involved in the control of locomotion and varies depending on gait speed and cognitive demand [31]. Cognitively impaired patients exhibit higher locomotor costs in dual-task conditions and an increased variability of time-related gait parameters [11]. These gait pattern changes even precede cognitive decline and might therefore be used as risk markers for early identification of dementia [12]. This condition has been previously termed motor cognitive risk syndrome [32]. The TUG test is also associated with cognitive functions, especially executive control, memory and processing speed [33]. Similarly, Pavlou et al. also depicted a correlation of cognitive function with FGA as well as dual task costs in chronic dizzy patients [34].

Brain imaging parameters such as focal cortical brain atrophy and white matter lesion load are well-known predictors for cognitive impairment [4]. Among the included visual atrophy scales, the GCA-F (addressing frontal lobe atrophy) is well-ascribed to higher cognitive functions including but not limited to language, working memory, problem solving, decision-making and behavior [35]. The MTA scale, on the other hand, assesses mesio-temporal atrophy, a well-established region for episodic and long-term memory and, more recently, for perception and attention [36]. There is also growing evidence highlighting the role of the insula in general cognitive functions such as language, perception, attention and working memory [4].

### Comparison with other cognition prediction models

The prediction models from this study performed comparably and even superior to other previously reported algorithms in the risk prediction of cognitive impairment, although differences in patient collectives limit a direct comparison. A gait feature-based model for detecting cognitive dysfunction in the elderly utilizing a single wearable inertia sensor yielded an AUC of 0.73–0.88 [37]. A standardized evaluation of multiple cognition prediction algorithms based on imaging data showed that the best AUC achieved was about 0.79 [38]. In comparison, our study revealed an AUC of 0.66 for a combination of gait parameters and an AUC of 0.71 for a set of semi-quantitative white-matter lesion and atrophy imaging parameters (Fig. 3A). A recently proposed machine learning algorithm for detecting cognitive impairment based on multiple features reflecting demographic, clinical, psychological and lifestyle aspects achieved an AUC of 0.73–0.83 [39, 40]. In our binary logistic regression model, the combination of different feature categories resulted in a superior predictive value (AUC of 0.84). It should be noted that we applied features to the model, which were already available from routine data sources and could be quantified without major data preprocessing. The proposed models therefore should be suitable for application in a clinical setting of risk prediction. A combination of feature sources was superior to single feature source for our cohort.

## Conclusions

The most important risk predictors of cognitive impairment in elderly dizzy patients, aside from the usual demographic factors, are postural instability, normal peripheral vestibular test results, impaired gait performance, higher frontal, insular and temporal focal brain atrophy, and white matter lesion load. We think that the proposed models may help clinicians to efficiently identify elderly patients with otherwise unexplained chronic dizziness at risk of DCI syndrome, to apply advanced cognitive testing for the differential diagnosis and appropriate neuropsychological training and drug treatment.

### Supplementary Information

Below is the link to the electronic supplementary material.

Supplementary file1 (DOCX 16 KB)

## Data Availability

The data supporting the conclusions of this article will be made available by the authors upon reasonable request to the corresponding author.
