# Supplementary material for: Evaluation of a multimodal diagnostic algorithm for prediction of cognitive impairment in elderly patients with dizziness
Source: J Neurol. 2024 May 3;271(7):4485–94. doi: 10.1007/s00415-024-12403-3 (PMC11233323; doi:10.1007/s00415-024-12403-3)
Supplement: Supplementary file 1 — Supplementary file1 (DOCX 16 KB) [file 415_2024_12403_MOESM1_ESM.docx]

|  | Prevalence | | Chi²-test (p-value corrected for age) |
| --- | --- | --- | --- |
|  | MoCA <25 (n=45) | MoCA $\geq$25 (n=55) |  |
| **Duration:** |  |  |  |
| Episodes | 8 (17.8%) | 20 (36.4%) | 0.091 |
| Persistent | 43 (95.6%) | 48 (87.3%) | 0.181 |
| **Character:** |  |  |  |
| Rotatory vertigo | 6 (13.3%) | 15 (27.3%) | 0.216 |
| Staggering dizziness | 19 (42.2%) | 27 (49.1%) | 0.094 |
| Drowsiness | 10 (22.2%) | 12 (21.8%) | 0.817 |
| Unsteady gait | 40 (88.9%) | 50 (90.9%) | 0.945 |
| >1 complaint | 27 (60%) | 40 (72.7%) | 0.281 |
| **Accompanying symptoms:** |  |  |  |
| Cochlear | 11 (24.4%) | 18 (32.7%) | 0.659 |
| Central signs | 29 (64.4%) | 26 (47.3%) | 0.226 |
| Falls | 21 (46.7%) | 21 (38.2%) | 0.486 |
| Nausea/vomiting | 3 (6.7%) | 7 (12.7%) | 0.589 |
| Photo-/Phonophobia | 4 (8.9%) | 2 (3.6%) | 0.235 |
| **Trigger:** |  |  |  |
| Changing head position | 7 (15.6%) | 11 (20%) | 0.838 |
| Quick head rotation | 4 (8.9%) | 13 (23.6%) | 0.110 |
| Orthostatic | 8 (17.8%) | 11 (20%) | 0.907 |
| Excercise/stress | 24 (53.3%) | 17 (30.9%) | 0.050 |
| Darkness/uneven ground | 18 (40.0%) | 36 (65.5%) | 0.031* |
| Foreign environment | 4 (8.9%) | 12 (21.8%) | 0.126 |
| Dependent of daytime | 6 (13.3%) | 10 (18.2%) | 0.480 |

**Supplementary table**: Detailed symptom characteristics in patient subgroups (MoCA <25 vs. ≥25).
